# Evolution of queen pheromone receptor tuning in four honeybee species (Hymenoptera, Apidae, Apis)

**DOI:** 10.1016/j.isci.2024.111243

**Published:** 2024-10-24

**Authors:** Julia Mariette, Julie Carcaud, Thierry Louis, Eleanor Lacassagne, Ilana Servais, Nicolas Montagné, Thomas Chertemps, Emmanuelle Jacquin-Joly, Camille Meslin, Frédéric Marion-Poll, Jean-Christophe Sandoz

**Affiliations:** 1 Evolution, Genomes, Behaviour and Ecology, IDEEV, Université Paris-Saclay, CNRS, IRD, 12 Route 128, 91190 Gif-sur-Yvette, France; 2 Sorbonne Université, INRAE, CNRS, IRD, UPEC, Université de Paris, Institute of Ecology and Environmental Sciences of Paris (iEES-Paris), Paris, France

**Keywords:** Entomology, Evolutionary biology, Molecular biology

## Abstract

Honeybees (genus: Apis) use a plethora of pheromones for intraspecific communication. The primary compound produced by the queen’s mandibular glands, 9-ODA, is involved in mating in all Apis species. It is the ligand of the most highly expressed olfactory receptor in males of *Apis mellifera*: *Amel*OR11. Putative orthologs are found in the genomes of other Apis species: *Apis dorsata*, *Apis florea*, and *Apis cerana*. Modeling of OR11 proteins shows high structure conservation except for *Aflor*OR11. Using heterologous expression in *Drosophila* and calcium imaging, a broad odorant screening revealed that all OR11 respond predominantly to 9-ODA, but also to secondary ligands, except *Aflor*OR11, which remains specific to 9-ODA. Secondary ligands were confirmed by optical imaging of male antennal lobes in *A. mellifera*. This work supports a conserved queen sex pheromone detection channel in honeybees, albeit with an extended response spectrum possibly playing a role in reproductive isolation among species.

## Highlights

• Four OR11 orthologs in honeybees (Apis sp.) share a similar 3D structure
• Honeybee olfactory receptors can be expressed in fruit fly olfactory neurons
• OR11 orthologs strongly respond to the honeybee queen sex pheromone 9-ODA
• OR11 orthologs exhibit responses to different sets of secondary ligands

## Graphical abstract

**Figure:**
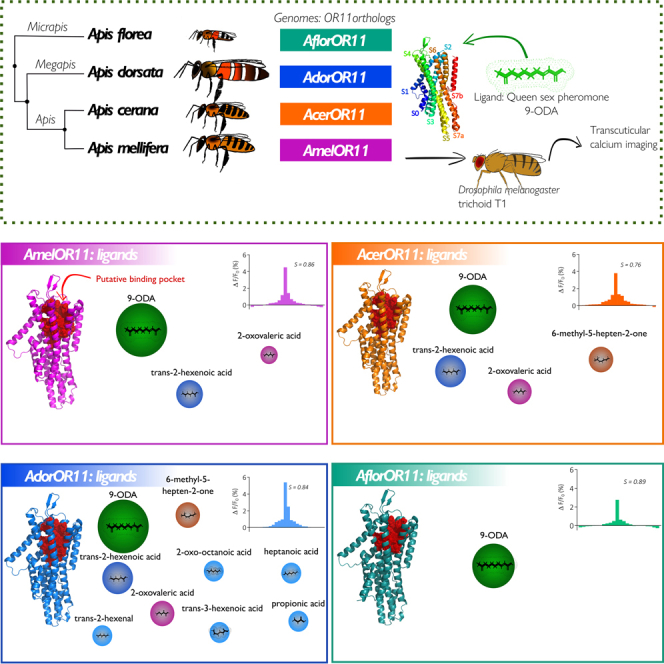

## Introduction

The mechanisms underlying speciation are fundamental to evolutionary biology and involve the establishment of barriers between populations of the same species, whether geographical, ecological, or reproductive.^1^^,^^2^ At an evolutionary scale, it is difficult to observe species undergoing speciation, but it is possible to understand some of the involved mechanisms by observing evidence of recent events enabling reproductive isolation or the gradual establishment of pre-zygotic reproductive barriers among closely related species.^1^^,^^3^^,^^4^^,^^5^^,^^6^^,^^7^^,^^8^ In insects, olfaction is often the predominant sensory modality for intersexual interactions, so the most commonly studied mechanism leading to reproductive isolation is the divergence of chemical communication via sex pheromones.^3^^,^^9^^,^^10^^,^^11^

Honeybees (Hymenoptera: Apidae, tribe Apini, genus Apis) are a group of eusocial bee species characterized by a set of remarkable features, such as the hexagonal structure of their combs made of wax, extreme multiple mating by queens, and the use of a complex communication system known as the dance language.^12^^,^^13^ The Apis genus, with its origin in Southeast Asia, is presently acknowledged to comprise at least nine well-described species, but up to fifteen species have been suggested.^14^^,^^15^^,^^16^ These species are distributed across three main lineages, corresponding to subgenera: the *Micrapis* or dwarf honeybees, the *Megapis* or giant honeybees, both open-nesting species, and the *Apis*, a group of cavity-nesting honeybees.^15^^,^^17^^,^^18^ In addition to their size, these species can be differentiated by a series of traits including the complexity of nest construction, division of labor, or their dance language, among others.^13^^,^^19^^,^^20^^,^^21^^,^^22^ The western honeybee, *Apis mellifera* (a cavity-nesting bee) has been the most studied species due to its apicultural and agricultural services and its wide distribution around the world.^23^^,^^24^ The other Apis species are all present in Asia and appear to be sympatric in many regions.^25^^,^^26^^,^^27^ Interestingly, all honeybees exhibit a very similar and remarkable mating behavior.^26^^,^^28^^,^^29^^,^^30^^,^^31^ During the mating season, when weather conditions are suitable, thousands of sexually mature males gather in the air and form so-called “drone congregations.”^25^^,^^32^^,^^33^^,^^34^ When a virgin queen joins the congregation, the drones are attracted by visual as well as olfactory cues (the queen sex pheromone) and enter into a scramble competition to mate with her.^35^ The queen mates with multiple drones, which die directly after copulation.^29^^,^^36^^,^^37^^,^^38^

Honeybee queens produce a range of pheromonal components within their mandibular glands. The term “Queen Mandibular Pheromone” (QMP) was created in *A. mellifera* for referring to the mixture of five compounds found in *mated* queens, which play a role on workers’ physiology and behavior.^39^ Among them, three primary compounds are universally shared among all Apis species: 9-oxo-(E)-2-decenoic acid (9-ODA) and two enantiomers, (R)- and (S)-9-hydroxy-(E)-2-decenoic acid (9-HDA). Variation in QMP composition is observed among Apis species: cavity-nesting bees include an additional compound, p-hydroxybenzoate (HOB), whereas open-nesting bees such as *Micrapis* and *Megapis* exclusively possess the aforementioned common compounds.^40^^,^^41^^,^^42^^,^^43^^,^^44^ The western honeybee, *A. mellifera*, exhibits a distinct QMP profile with the inclusion of a fifth compound, 4-hydroxy-3-methyoxyphenylethanol (HVA). In *virgin* queens, the presence and relative proportions of these compounds differ from mated queens, but some QMP compounds also play a role as sex pheromone. The most prominent, 9-ODA, is produced in high quantity by virgin queens and is known to attract males in all nine recognized species of the genus Apis.^14^^,^^25^^,^^26^^,^^40^^,^^41^^,^^42^^,^^45^^,^^46^^,^^47^^,^^48^^,^^49^ It is thus considered as the main sex pheromone in honeybees. However, some other compounds of the mandibular glands are thought to play a complementary role in honeybee reproduction. Thus, (R)- and (S)-9-HDA and a compound also produced in high amounts in the queen’s mandibular glands, 10-HDA, have been identified as having a role in attracting drones in *Apis mellifera* and *Apis florea*.^46^^,^^50^

The main role of drones is to mate with virgin queens, and evolution has led their olfactory system to become specialized for mating.^31^ In insects, odorants (including pheromones) are detected by transmembrane proteins called olfactory receptors (ORs), expressed by olfactory sensory neurons (OSNs), that are located within sensilla on the antennae. These neurons project to spheroidal units—the glomeruli—in the first olfactory center of the brain, the antennal lobe (AL). After processing by local networks, neural information is further transmitted to two higher-order centers, the mushroom bodies and the lateral horn. Commonly in insects, odorants with a high ecological relevance—especially sex pheromones—are processed by dedicated and relatively insulated neural pathways in the brain, allowing them to elicit rapid and specific behavioral responses. By contrast, other odorants are detected by multiple ORs and follow a combinatorial coding principle that increases coding capacity and allows insects to code for complex odorant blends.^51^^,^^52^

To date, most progress in elucidating the neural pathway for processing sex pheromone information in honeybees has been made in *A. mellifera.* Compared with workers, *A. mellifera* drones have larger antennae and ∼6 times more sensilla placodea and OSNs reviewed in Refs.^31^^,^^53^ Four of the ∼170 ORs contained in the genome of this species are strongly overexpressed in the drone antenna compared with that of workers.^54^^,^^55^ One of these receptors, *Amel*OR11, is expressed in ∼35% of drone OSNs^56^ and has been shown to respond to 9-ODA.^54^ The genomes of three other species of the Apis genus have been fully sequenced and annotated, and interestingly, they all present orthologs of *Amel*OR11: the Asian honeybee *Apis cerana* (*Acer*OR11), the giant honeybee *Apis dorsata* (*Ador*OR11), and the dwarf honeybee *Apis florea* (*Aflor*OR11).^57^^,^^58^^,^^59^ Sex dimorphism in the honeybee olfactory system is further observed by the existence of greatly enlarged glomeruli in the AL of drones, called macroglomeruli (MGs).^60^^,^^61^^,^^62^ First described in male moths,^63^^,^^64^^,^^65^ macroglomeruli receive the axon terminals of the numerous OSNs tuned to sex pheromones. The macroglomeruli found in the male ALs of these four species of *Apis* strongly suggest the presence of specialized neural pathways for processing sex-pheromones. The largest macroglomerulus, MGb, was shown to be specifically tuned to 9-ODA in the *Apis mellifera* male AL where it is called MG2,^62^ and homologs of this macroglomerulus are found in *A. cerana*, *A. florea*, and *A. dorsata* males.^60^ An attractive hypothesis would be to deduce that a processing pathway for queen pheromone compound, 9-ODA, would be evolutionary conserved within the *Apis* genus*.* However, the protein sequences of the different OR11 genes differ by several residues, and no data are yet available on the ligands of OR11 in *A. cerana*, *A. florea*, and *A. dorsata* or any other honeybee species.

The present study is thus dedicated to unraveling the odorant tuning of honeybee OR11 orthologs. To address this question, we expressed the four OR11 genes in *Drosophila melanogaster* within olfactory sensory neurons (OSNs) used as “empty neurons”^66^ and measured the responses of OR11-expressing OSNs to a wide range of floral and honeybee compounds. Our study establishes that all OR11 are tuned to 9-ODA and respond to this main ligand with different intensities. In addition, we show that they respond to a range of secondary ligands, with the exception of *Apis florea* OR11, which was uniquely tuned to 9-ODA. Our data therefore establish the presence of a sex pheromone pathway common among Apis species and yet whose odorant tuning varies across species.

## Results

### Heterologous expression of Apis ORs in *Drosophila* olfactory neurons

**Figure 1:**
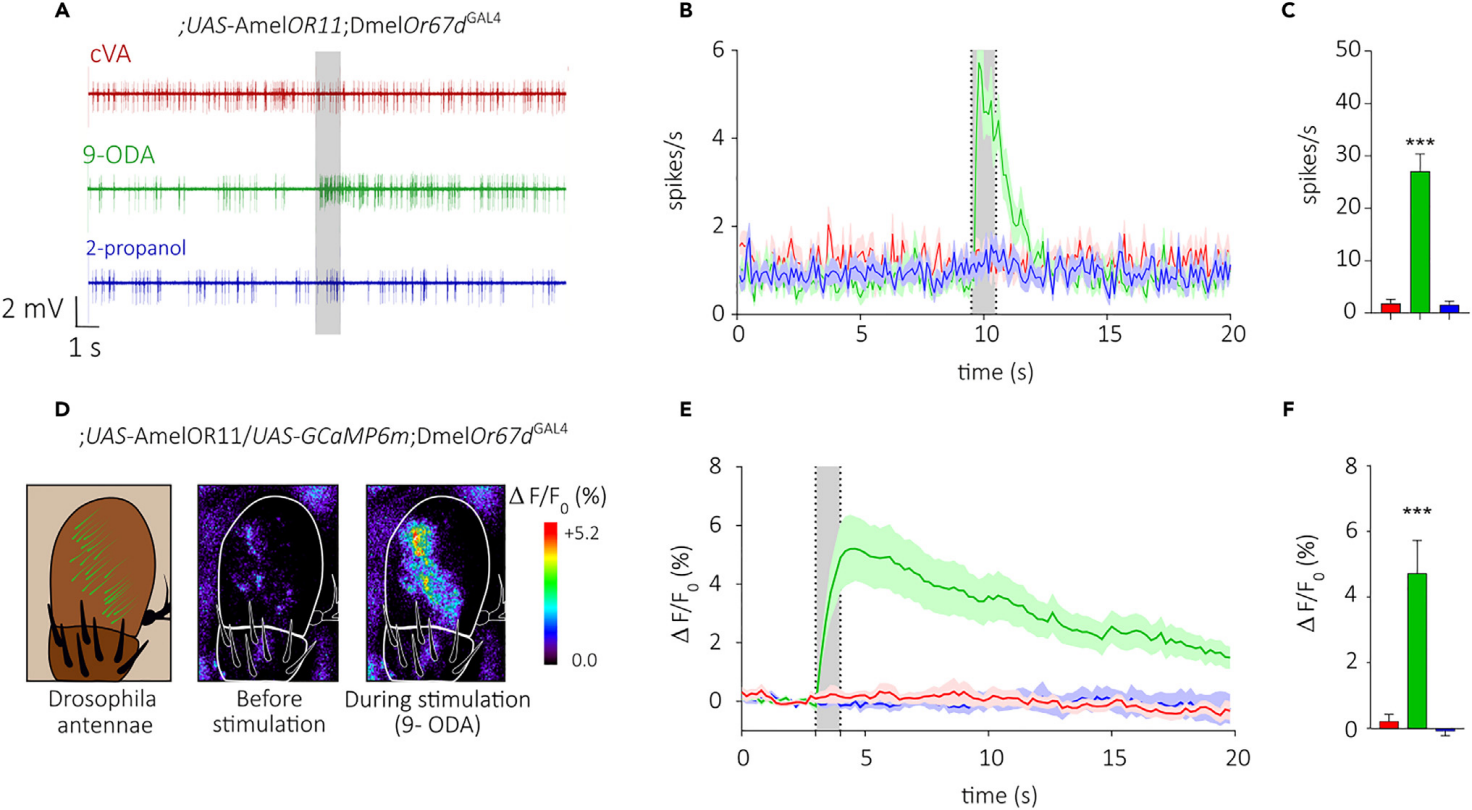
Responses of *Drosophila melanogaster* at1 OSNs expressing *Apis mellifera* OR11 (A) Representative recordings from an at1 OSN of a *w; UAS-AmelOR11; Or67d*^*GAL4*^ fly. The gray area represents the odorant stimulation (1 s). Spike frequency increased when 9-ODA was presented. (B) Instantaneous action potential frequency over time (plotted as mean ± SEM spikes.s^−1^, *n* = 12) for each stimulus (cVA, 9-ODA, 2-propanol). (C) Mean response to the odorants (mean ± SEM in spikes.s^−1^, ANOVA for repeated measurements, Greenhouse-Geisser correction and Dunnett’s multiple comparison test, *n* = 12, ∗∗∗*p* < 0.0001). (D) From left to right: a representation of the third segment of a *Drosophila* antenna, the funiculus, with the location of trichoid at1 sensilla in green; measured calcium activity in a *w*; *UAS-AmelOR11/UASGCaMP6m; Or67d*^*GAL4*^ fly *before* or *during* odorant stimulation with 9-ODA. Relative fluorescence changes are presented in a false color code from dark blue (no response) to red (maximum) (ΔF/F_0_%). (E) Average changes in fluorescence over time (mean ± SEM, *n* = 16). The gray area represents the odorant stimulation (1 s). (F) Mean amplitude of the calcium response per odorant (mean ± SEM, *n* = 16) (Friedman test, Dunn’s multiple comparison test, ∗∗∗*p* < 0.001).

As a first step, we expressed the queen pheromone receptor *Amel*OR11^54^ within at1 OSNs of the fruit fly, thereby replacing the endogenous receptor DmelOR67d (*w; UAS-GCaMP6m/UAS-xOR11; Or67d*^*GAL4*^). To validate the fruit fly line, we recorded odor-evoked responses from at1 neurons using SSR (Single Sensillum Recording). Two odorants were presented: 9-ODA, the known ligand of *Amel*OR11,^54^ and cVA, the ligand of *Dmel*OR67d. As expected, in transformed flies, at1 OSNs responded to 9-ODA, but not to cVA nor to the solvent alone (Figures 1A–1C).

After establishing the efficiency of honeybee OR expression within the *Drosophila* empty neuron system, we tested whether the use of transcuticular calcium imaging was efficient for the functional analysis of honeybee ORs, as shown recently for moth ORs.^67^ Flies expressing simultaneously *Amel*OR11 and the calcium sensor GCaMP6m were generated and subjected to the same olfactory stimulations as in SSR (Figure 1D). In agreement with the SSR results, 9-ODA triggered a strong calcium response from the imaged at1 neurons, whereas cVA and 2-propanol induced no response (Figures 1E and 1F). These experiments show that honeybee ORs can be expressed in *Drosophila* at1 OSNs and that their activity can be recorded with both SSR and transcuticular calcium imaging.

### Structure and function similarities between the four Apis OR11 orthologs

**Figure 2:**
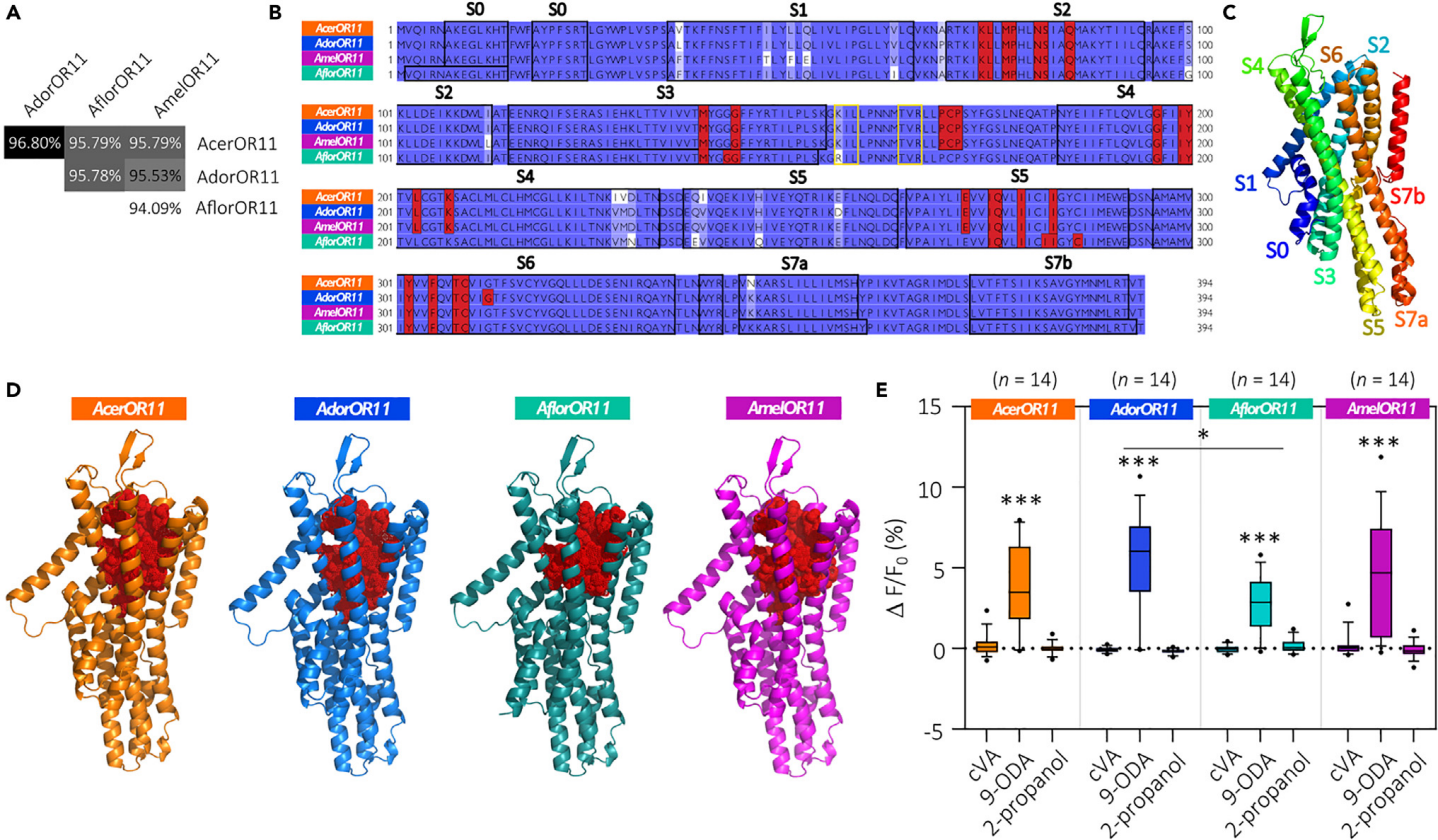
Comparison of protein sequences, structures, and odorant response profiles of the four OR11 (A) Percentage of identity between the four ortholog OR11 nucleotide sequences. (B) Protein sequences alignment of *Acer*-, *Ador*-, *Aflor*-, and *Amel*-OR11. Sequence identity similarities are represented from identical (blue) to different (in white). Amino acids highlighted in red are in the predicted binding pocket and located within 5 Å of the nine poses of the queen pheromone compound 9-ODA. Black boxes delineate the helical subunits (from S0 to S7b), and yellow boxes between the 3^rd^ and 4^th^ helices represent the β-sheets. (C) Predicted structure of *Amel*OR11 in side view. Helices S0 to S7b are colored according to a rainbow spectrum from the N-terminus (in blue) to the C-terminus (in red). (D) Molecular docking of the binding pockets for the four OR11 receptors. Amino acids shown in red are within 5 Å of one of the nine putative poses of 9-ODA and are thought to interact with this ligand by hydrogen bonds. Red residues involved in the binding pockets are shown as stick structure, surrounded by dots that indicate electron clouds. (E) Boxplots of calcium responses measured for the OR11 orthologs using transcuticular calcium imaging of the *Drosophila* antenna (whiskers are 10–90 percentiles, Friedman test for comparison among stimuli, Dunn’s multiple comparison test, ∗∗∗*p* < 0.001; Kruskal-Wallis test for comparison between ORs, Dunnett test for multiple comparison test, ∗*p* < 0.05).

The protein sequences of the *Apis mellifera* OR11 orthologs in *A. cerana*, *A. dorsata*, and *A. florea* were aligned, and structural modeling was performed based on the protein sequence, using *A. mellifera* OR11 as template. The alignment of nucleotide sequences revealed very similar genes (between 94.09% and 96.80% identity) with only a few nucleotide differences (Figure 2A). Phylogenetic analysis of protein sequences based on the percentage of identity among OR11 proteins revealed a strong resemblance between *A. cerana* and *A. dorsata* (98,48%), both differing moderately from *A. mellifera* (97,46%) and slightly more from *A. florea* OR11 (96,95%) (Figure 2A). All OR11 proteins have an identical conformation, with identical length and the same set of helical subunits, although slight differences were observed in four helices (S0, S3, S7a, and S7b) for *Aflor*OR11 (Figure 2B). Most amino acid substitutions were found within the helices (Figures 2B and 2C). However, we identified two putative binding pockets for *Acer*-, *Ador*-, and *Amel*OR11 and three putative binding pockets for *Aflor*OR11, which differed from the other OR11 (Table S2). The location of the binding sites was almost identical for *Acer*-, *Ador*- and *Amel*OR11, but for *Aflor*OR11, the binding site was located slightly lower from the top of the protein and was smaller, involving 23 residues against 26 for *Acer-* and *Amel*OR11 and 27 for *Ador*OR11 (Figure 2D). The queen pheromone compound 9-ODA can bind residues in nine different poses within the binding pocket, and amino acids located within 5 Å of the nine poses of the queen pheromone were highly similar between *Acer*-, *Ador*-, and *Amel*OR11 (Figures 2A and 2D), yet residue G312 appeared involved in the binding pocket of *Ador*OR11 but not of *Acer*OR11 and *Amel*OR11. *Aflor*OR11 was also very similar but four amino acids differed (Figures 2B–2D). We observed that three residues, P169, C170, and P171, present in the second extracellular loop, seem to interact with the ligand for *Acer*OR11, *Ador*OR11, and *Amel*OR11 but not for *Aflor*OR11, as well as some residues in the fourth and fifth helices (Figures 2B and 2D). In summary, although the four OR11 protein structures appeared highly conserved across species, a number of mutations could induce differences in odor tuning among the receptors, especially concerning *Aflor*OR11^9^ (Figure 2D).

**Figure 3:**
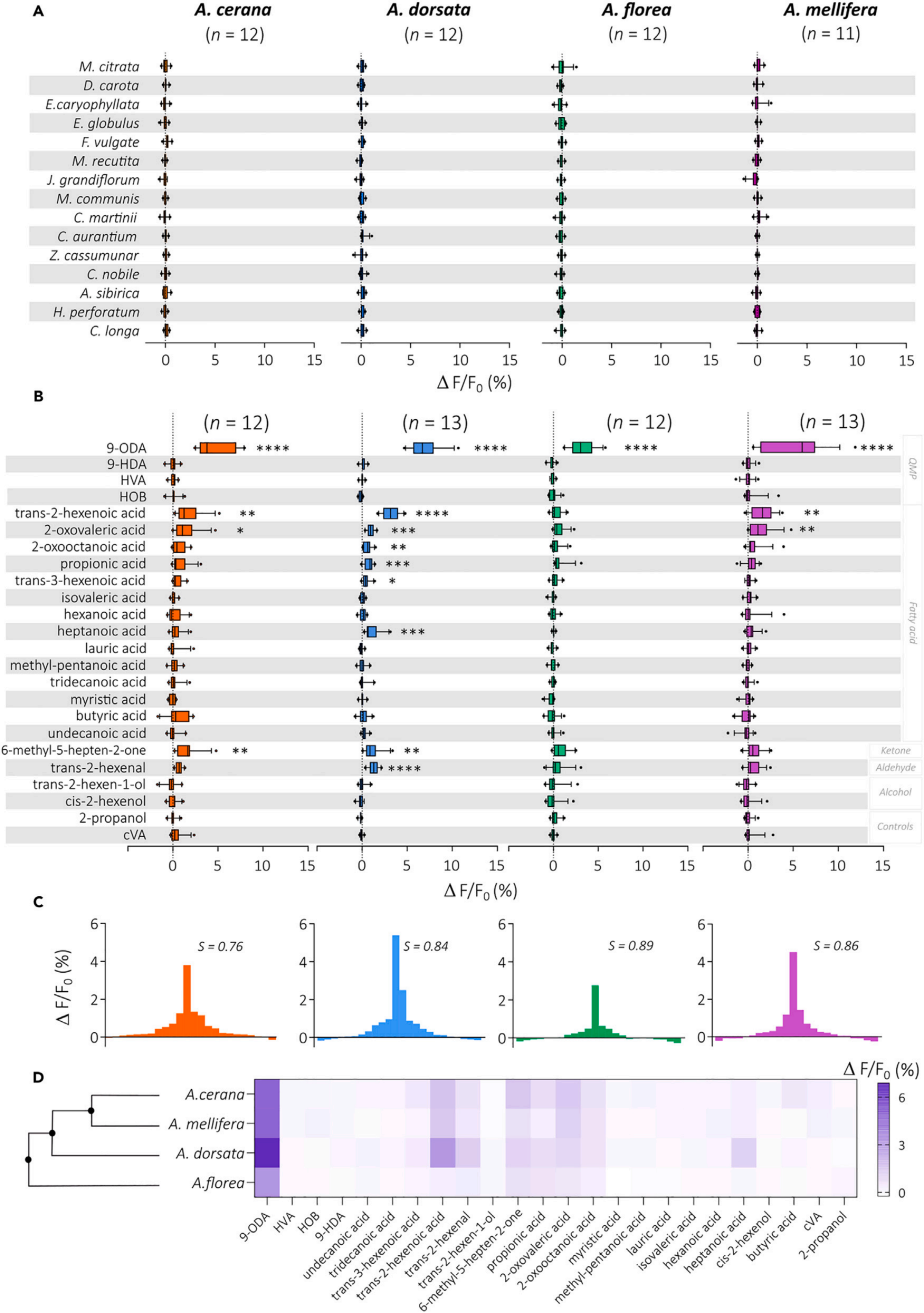
The four OR11 orthologs are narrowly tuned to 9-ODA but respond to some secondary ligands (A) Boxplot of calcium responses (whiskers are 10–90 percentiles) to 15 essential oils (all diluted in mineral oil, 1:100) recorded from at1 sensilla (*Acer*OR11: *n* = 12, *Ador*OR11: *n* = 12, *Aflor*OR11: *n* = 12, *Amel*OR11: *n* = 11; Friedman Test, Dunn’s multiple comparison test). (B) Boxplot (whiskers are 10–90 percentiles) of calcium responses to 22 odorants (QMP + odorants with similar chemical properties, all diluted in 2-propanol) (means ± SEM; *Acer*OR11: *n* = 12, *Ador*OR11: *n* = 13, *Aflor*OR11: *n* = 12, *Amel*OR11: *n* = 13; Friedman Test, Dunn’s multiple comparison test, ∗*p* < 0.05, ∗∗*p* < 0.01, ∗∗∗*p* < 0.001, ∗∗∗∗*p* < 0.0001). (C) Distribution of mean responses of each OR11 to the panel of 22 odorants (controls included). The tuning breadth is quantified by the sparseness value of the distribution (S).^68^^,^^69^ (D) Dendrogram on the left and heatmap of the responses to the panel of odorants in (B) for each OR11.

Given the similarity in their sequences and predicted structures, we first investigated if *Acer*OR11, *Ador*OR11, and *Aflor*OR11 also respond to the known ligand of *Amel*OR11, 9-ODA (Figure 2E). As before, 9-ODA and the two controls, 2-propanol and cVA, were presented. All three orthologs of *Amel*OR11 showed a strong and highly significant response to 9-ODA. Interestingly, although the experiments were performed simultaneously and in the same conditions, a significant difference was observed among the amplitudes of the responses to 9-ODA, with the strongest response for *A. dorsata* and the lowest for *A. florea*. Such differences were reproducible and were observed in all our later experiments (Figure 3B).

### *Amel*OR11 and its orthologs respond to fatty acids

The previous experiment showed that the four OR11 orthologs respond to 9-ODA. Although these receptors are pheromone receptors and are thus expected to show very specific responses,^54^ little is known about other possible ligands. We next endeavored to determine the specificity of these receptors and to discover such ligands. As a first step, we applied a screening strategy based on the presentation of numerous complex odorant mixtures, offering a broad sample of possible ligands. Thus, the queen pheromone receptor orthologs were exposed to a panel of 15 essential oils (see composition in Table S1). This panel presented a wide variety of chemical compounds (esters, terpenes, alcohols, aldehydes, etc.). This broad screening did not elicit any remarkable activity in any of the tested OR11 (Figure 3A). We conclude that none of the OR11 orthologs responds to the set of floral odorants contained in our essential oils.

To further explore the response spectra of OR11, we composed a set of odorants based on their structural similarity with the ketoacid 9-ODA as well as a previous electrophysiological study (Vareschi, 1971—see Table 1). Vareschi (1971) used a cross-adaptation strategy, in which two consecutive stimulations with different odorants are considered to activate the same neuron type if adaptation is observed. Doing so, he described 10 different “units” (which would correspond today to OSNs carrying a particular OR) in the antenna of honeybees. One of these units responded to 9-ODA (“queen substance” in Vareschi 1971) as well as a list of about 20 other, chemically similar, compounds. We also presented other compounds from the queen mandibular pheromone (the two enantiomers (R)- and (S)-9-HDA, HVA, and HOB). As before, strong responses were observed to the queen pheromone 9-ODA for the four OR11s, with the same differences in magnitude between species (strongest response for *A. dorsata*, lowest response for *A. florea*). Interestingly though, significant responses to several other stimuli were also observed (Figure 3B). *Amel*, *Acer*, and *Ador*OR11 all responded significantly to two fatty acids in the panel: *trans*-2-hexenoic acid and 2-oxovaleric acid. *Acer*OR11 responded to a third secondary ligand: 6-methyl-5-hepten-2-one. *Ador*OR11 showed responses to eight stimuli in the panel. They included the three secondary ligands of *Acer*OR11 plus five other compounds: heptanoic acid, propionic acid, trans-2-hexenal, 2-oxooctanoic acid, and *trans*-3-hexenoic acid. Lastly, *Aflor*OR11 was only activated by 9-ODA, and no other ligand was found.

**Table 1:** List of compounds tested on the four OR11 orthologs of the honeybee

Molecule	Family	Molecular weight	Formula	
9-ODA	Fatty acid	184.23	C_10_H_16_O_3_	*QMP*
9-HDA	Fatty acid	186.25	C_10_H_18_O_3_	
HOB	Ester	152.15	C_8_H_8_O_3_	
HVA	Benzene/Phenols	168.19	C_9_H_12_O_3_	
*Cis*-2-hexen-1-ol	Alcohol	100.16	C_6_H_12_O	*Compounds selected for queen-pheromone-like conformation*
*Trans*-2-hexen-1-ol	Alcohol	100.16	C_6_H_12_O	
6-Methyl-5-hepten-2-one	Ketone	126.2	C_8_H_14_O	
*Trans*-2-hexenal	Aldehyde	98.14	C_6_H_10_O	
Butyric acid	Fatty acid	88.11	C_4_H_8_O_2_	
Heptanoic acid	Fatty acid	130.18	C_7_H_14_O_2_	
Hexanoic acid	Fatty acid	116.16	C_6_H_12_O_2_	
Isovaleric acid	Fatty acid	102.13	C_5_H_10_O_2_	
Lauric acid	Fatty acid	200.32	C_12_H_24_O_2_	
4-Methyl-pentanoic acid	Fatty acid	116.16	C_6_H_12_O_2_	
Myristic acid	Fatty acid	228.37	C_14_H_28_O_2_	
2-Oxooctanoic acid	Fatty acid	158.19	C_8_H_14_O_3_	
2-Oxovaleric acid	Fatty acid	116.11	C_5_H_8_O_3_	
Propionic acid	Fatty acid	74.08	C_3_H_6_O_2_	
*Trans*-2-hexenoic acid	Fatty acid	114.14	C_6_H_10_O_2_	
*Trans*-3-hexenoic acid	Fatty acid	114.14	C_6_H_10_O_2_	
Tridecanoic acid	Fatty acid	214.34	C_13_H_26_O_2_	
Undecanoic acid	Fatty acid	186.29	C_11_H_22_O_2_	

To evaluate the proximity between response patterns of the four OR11s, odorant response spectra were compared. Based on the patterns of responses measured in this experiment, we tentatively measured each receptors’ tuning breadth using a sparseness measure of response distribution^68^^,^^69^ (Figure 3C). The values of sparseness are bounded between 0 and 1, thus a low S value indicates a broad tuning of the receptor (i.e., a generalist receptor) while a value of 1 indicates a narrow tuning (i.e., a specialist receptor). All four receptors showed very high sparseness values (0.76–0.89). *Acer*OR11 showed the lowest sparseness (S = 0.76), meaning that its response spectrum was the broadest, whereas *Aflor*OR11 showed the highest sparseness, 0.89, and consequently is considered the most specific receptor. The similarity relationships among the response patterns were assessed using a cluster analysis^70^ (Figure 3D). The dendrogram obtained matches the species phylogeny and places *Aflor*OR11 as the most different from the other receptors (Figure 3D; Smith^14^).

### Sensitivity of OR11 orthologs to 9-ODA and secondary ligands

**Figure 4:**
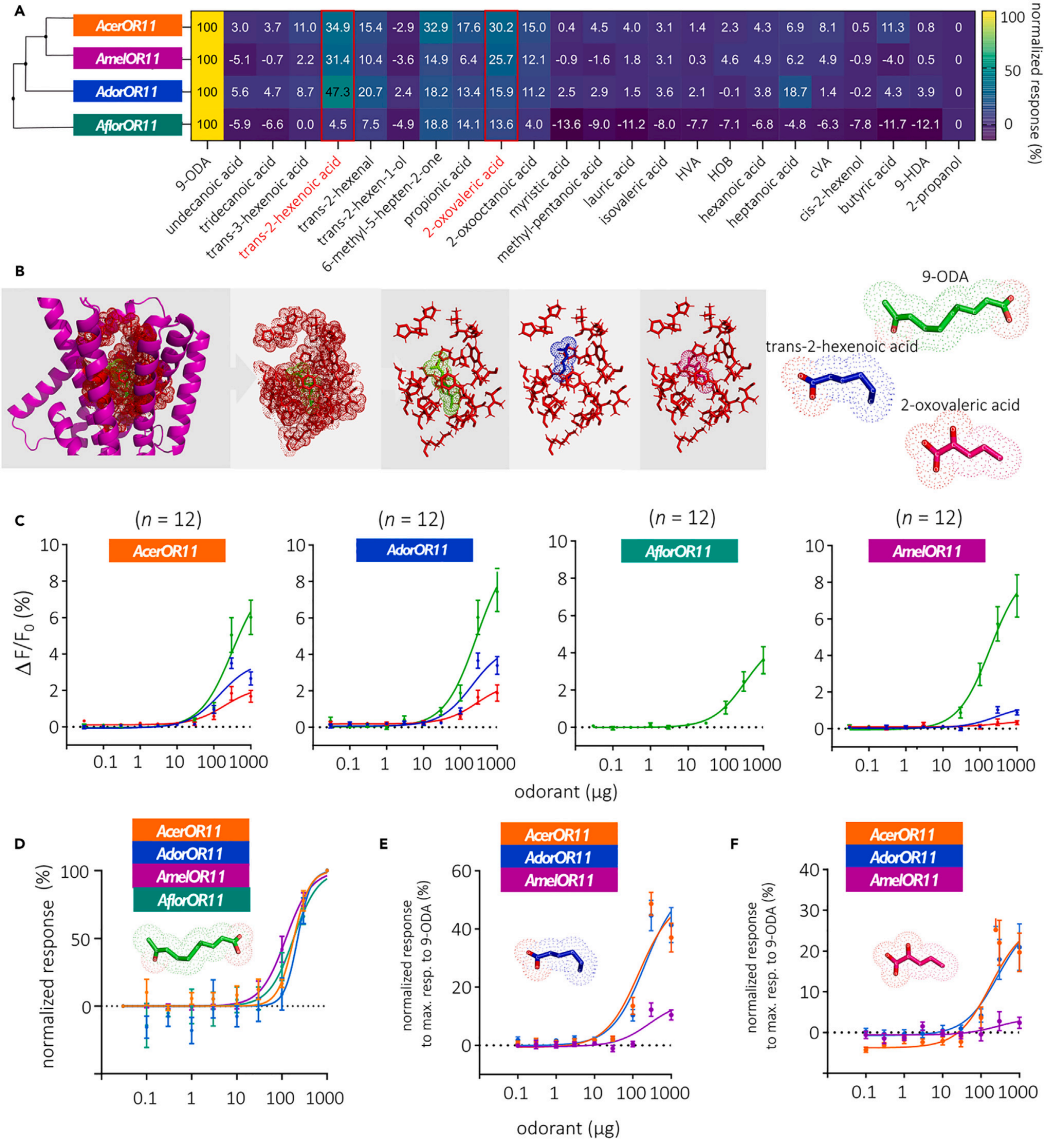
Different sensitivity depending on the ligand for the four OR11 orthologs (A) Heatmap of normalized responses to the panel of odorants in Table 1 for each OR11 (maximum amplitude normalized at 100% and minimum response at 0%). (B) *From left to right*: molecular docking mapping representation of the *Amel*OR11 binding pocket (red residues) interacting with the three ligands, 9-ODA (green), *trans*-2-hexenoic acid (blue), and 2-oxovaleric acid (pink) (dots represent valence shell electrons). *Right:* chemical representation of the three ligands, 9-ODA in green, *trans*-2-hexenoic acid in blue, and 2-oxovaleric acid in pink; oxygen atoms are shown in red; dots represent valence shell electrons. (C) Dose-response curves of *Acer*OR11, *Ador*OR11, *Aflor*OR11, and *Amel*OR11 for the three odorants (9-ODA, *trans*-2-hexenoic acid, 2-oxo-valeric acid) diluted in 2-propanol, shown as the 0 μg dose (plotted as mean ± SEM, *n* = 12 in each case). (D) Normalized dose-response curves (plotted as mean ± SEM) of *Acer*OR11, *Ador*OR11, *Aflor*OR11, and *Amel*OR11 for 9-ODA, response to the solvent considered as 0% and response at the highest dose as 100%. (E and F) Responses to *trans*-2-hexenoic acid (E) and 2-oxo-valeric acid (F) after normalization with respect to the maximum response to 9-ODA for the three OR: *Acer*OR11 (orange), *Amel*OR11 (magenta), and *Ador*OR11 (blue) (plotted as mean ± SEM).

We next studied the sensitivity of the OR11 orthologs to their ligands: the main ligand, 9-ODA, as well as two fatty acids, *trans*-2-hexenoic acid and 2-oxovaleric acid, which appeared as the best secondary ligands shared among *Amel*OR11, *Ador*OR11, and *Acer*OR11 (see normalized response heatmap, Figure 4A). These last two compounds were not tested on *Aflor*OR11, as the previous experiment demonstrated that *Aflor*OR11 does not respond to them (Figure 3B). Using molecular docking with these new ligands, we observed that they potentially bind at a similar position and interact with similar residues as 9-ODA (Figure 4B). This can be explained by the similar chemical structure of these compounds compared with 9-ODA (Figure 4B).

We analyzed the sensitivity of the four receptors to 9-ODA (Figures 4C and 4D). All receptors showed responses that increased with the dose of 9-ODA, reaching different maximal intensities at the highest dose, as observed earlier. We initially compared response amplitudes for each dose between OR11s. We observed at 30 μg a significantly lower response of *Aflor*OR11 compared to *Ador*OR11 (Kruskal-Wallis test, *p* = 0.02) and at 100 μg, responses of *Acer*OR11 and *Aflor*OR11 were significantly lower than that of *Amel*OR11 (*p* = 0.01 and *p* = 0.008). At high doses, we found that the response of *Aflor*OR11 was significantly lower than for the three other OR11s at 300 μg and lower than for *Ador*OR11 and *Amel*OR11 at 1,000 μg (*p* < 0.001; see Table S3). When normalizing the data to each receptor’s maximal response, the four curves closely overlapped (Figure 4D). *Acer*OR11 responses to 9-ODA became significant at a dose of 30 μg. *Ador*OR11, *Amel*OR11, and *Aflor*OR11 showed significant responses at 100 μg and above. To compare the sensitivity of the four receptors, we calculated their EC_50_ and found remarkably similar values (*Acer*OR11: 174.8 ± 26.05 μg; *Ador*OR11: 173.4 ± 22.22 μg; *Amel*OR11: 160.5 ± 28.46 μg; *Aflor*OR11: 175.8 ± 29.59 μg, Kruskal-Wallis test, *p* = 0.98). The steepness of each curve, measured as the Hill coefficient, was also highly similar among receptors (*Acer*OR11: 4.71 ± 1.76; *Ador*OR11: 6.98 ± 2.83; *Amel*OR11: 2.31 ± 0.24; *Aflor*OR11: 13.04 ± 8.02, Kruskal-Wallis test, *p* = 0.22). Thus, the four OR11 orthologs showed similar dose-response curves to 9-ODA and consequently, a similar sensitivity to this ligand.

We then analyzed the sensitivity of *Acer*OR11, *Ador*OR11, and *Amel*OR11 to the two secondary ligands, *trans*-2-hexenoic acid and 2-oxo-valeric acid (Figures 4C–4F). *Acer*OR11 (*n* = 12) displayed a higher sensitivity to *trans*-2-hexenoic acid than to 2-oxo-valeric acid (Friedman Test, *p* < 0.0001). Indeed, we observed a significant response to *trans*-2-hexenoic acid from 100 μg (Dunn’s test, *p* = 0.0003), whereas significant responses to 2-oxovaleric acid started at 300 μg (*p* = 0.007). *Ador*OR11 (*n* = 12) showed similar sensitivity to the two odorants and displayed a significant response from 100 μg (*p* < 0.01). *Amel*OR11 (*n* = 10) showed the lowest sensitivity to the two secondary ligands. Responses to both fatty acids became significant at 300 μg only (*p* = 0.01). The comparison of response amplitudes at each dose revealed a significantly lower response of *Amel*OR11 to *trans*-2-hexenoic acid at 100, 300, and 1,000 μg (Kruskal-Wallis test, *p* < 0.01). However, no differences were observed in the responses of the different OR11s to 2-oxo valeric acid (*p* > 0.05).

As before, we calculated EC_50_ to compare the sensitivity of the three receptors and discovered a lower sensitivity of *Amel*OR11 to secondary ligands. EC_50_ to *trans*-2-hexenoic acid were significantly different between *Acer*OR11 (105.8 ± 9.62 μg) and *Amel*OR11 (239.2 ± 72.06 μg), *Acer*OR11 appearing more sensitive to this ligand (Kruskal-Wallis test, *p* = 0.01). However, no significant difference was noted between EC_50_ of *Ador*OR11 (124.2 ± 9.58 μg) and *Amel*OR11 (239.2 ± 72.06 μg; *p* = 0.23). The EC_50_ values for 2-oxovaleric acid were significantly different between *Acer*OR11 (208.5 ± 39.57 μg) and *Amel*OR11 (84.26 ± 15.52 μg), with surprisingly higher sensitivity for *Amel*OR11 (*p* = 0.04). However, EC_50_ between *Ador*OR11 (139.3 ± 28.55 μg) and the two others did not differ (*p* = 0.22). The steepness of each curve was also calculated (Hill coefficient) but no differences between OR11s were observed for the two ligands (*p* = 0.53). We conclude that although the different OR11 orthologs showed the same sensitivity to 9-ODA, their sensitivity to the secondary ligands differed.

To compare amplitudes of responses to secondary ligands between OR11s, we normalized them according to responses to 9-ODA (Friedman test, *p* < 0.001; Figures 4E and 4F). For *trans*-2-hexenoic acid, we observed that *Amel*OR11 is significantly less sensitive than *Ador*OR11 and *Acer*OR11 from 30 μg upward (Dunn’s test, *p* < 0.01). For 2-oxovaleric acid, significant differences between *Amel*OR11 and the two other OR11s were observed from 300 μg upward (*p* < 0.01). *Amel*OR11 appeared more narrowly tuned to 9-ODA than the two other OR11, especially for *trans*-2-hexenoic acid for which *Amel*OR11 sensitivity was lower than that of the two other receptors.

### Response to a secondary ligand of *Amel*OR11 in the drone antennal lobe

**Figure 5:**
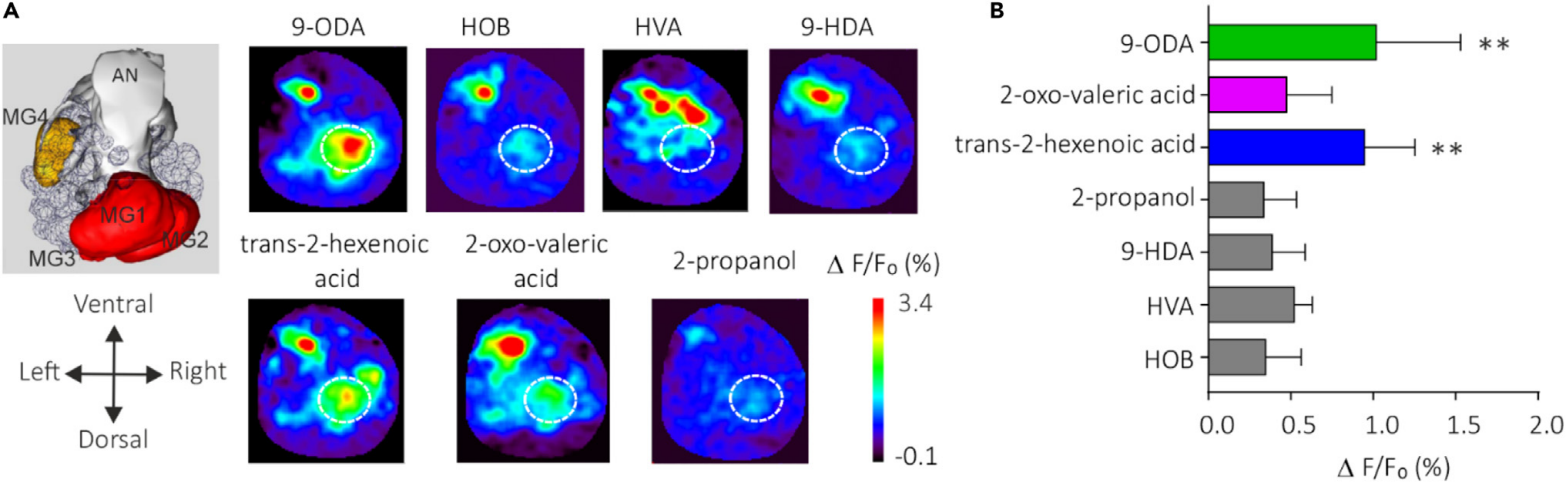
Odor-induced calcium signals in macroglomerulus 2 of male *Apis mellifera* (A) Left: maps of male right antennal lobe and location of macroglomeruli (MGs) and antennal nerve (AN). Right: calcium activity maps in the male *Apis mellifera* AL evoked by QMP compounds and secondary ligands (all diluted in 2-propanol). Relative fluorescence changes are presented in a false color code from dark blue (no response) to red (maximum) (ΔF/F_0_%). The location of MG2 is shown with a white dashed line. (B) Mean amplitudes of calcium responses (± SEM) to QMP compounds and two secondary ligands (9-ODA in green, *trans*-2-hexenoic acid in blue, and 2-oxo-valeric acid in pink) (*n* = 5, ∗∗: *p* < 0.01).

We found secondary ligands for three OR11 orthologs in the genus *Apis*, using heterologous expression in the *Drosophila melanogaster* OSNs followed by transcuticular calcium imaging. To assess the validity of these secondary ligands in honeybees, we chose to test whether they activate the antennal lobe in the brain of *Apis mellifera* males. As 9-ODA is known to activate an enlarged glomerulus, macroglomerulus MG2 in this species,^62^ we used *in vivo* calcium imaging to record odor-evoked responses in the antennal lobe of *A. mellifera* drones (Figure 5A). We tested the two secondary ligands of *Amel*OR11 (at 100 μg: *trans*-2-hexenoic acid and 2-oxovaleric acid), QMP compounds (at 250 μg: 9-ODA, 9-HDA, HVA, and HOB) and the solvent, 2-propanol. We observed a strong response to 9-ODA within MG2, 1.01 ± 0.51%, as expected. We also observed a significant response to *trans*-2-hexenoic acid in comparison to the solvent (0.94 ± 0.30, Figure 5B). However, even if a slight response appeared in some activity maps, the intensity of the calcium signals in response to 2-oxo-valeric acid (0.47 ± 0.27%) was not significantly higher than the control (0.33 ± 0.19%). We conclude that the strongest secondary ligand observed for OR11 in *A. mellifera* induces significant MG2 responses in the antennal lobe of this species.

## Discussion

In the present work, we studied odor-evoked response profiles of OR11 orthologs from four honeybee species: *A. cerana*, *A. dorsata*, *A. florea*, and *A. mellifera*. They were expressed within OSNs of trichoid sensilla at1 in *Drosophila melanogaster.* Using single-sensillum electrophysiological recordings and transcuticular calcium imaging of the fruit fly antenna, we confirmed that *Amel*OR11 is tuned to 9-oxo-(E)-decenoic acid (9-ODA), the major component of the queen mandibular pheromone.^54^ We discovered that the three other OR11s, *Acer*OR11, *Ador*OR11, and *Aflor*OR11, also strongly respond to 9-ODA. This result fits with the high similarity in the protein sequences of these receptors, which all appeared to have a similar binding pocket. In addition, for the three receptors *Acer*OR11, *Ador*OR11, and *Amel*OR11, significant but less intense responses were also observed to odorants that share some similarities with 9-ODA in their chemical structure. The three receptors exhibited similar response spectra with slight differences, with *Acer*OR11 and *Ador*OR11 exhibiting the broadest spectra. Dose-response analyses revealed similar sensitivities among species to the main ligand, 9-ODA, but different sensitivities to one of the secondary ligands, *trans*-2-hexenoic acid. Lastly, similar to 9-ODA, this latter ligand activated macroglomerulus 2 within the antennal lobe of male *A. mellifera*, thereby corroborating what was observed in a heterologous expression system.

The protein sequences of the four OR11 are highly similar, only differing by a few amino acids, which do not appear to affect their main odorant tuning. Modeling of their 3D structure showed similar transmembrane domains as well as extra- and intra-cellular loops. The amino acids that putatively compose the binding pocket were also highly similar but not exactly the same. Only *Aflor*OR11 appeared to display differences in structure compared to the three other receptors. Interestingly, *A. florea* belongs to the *Micrapis* lineage, the most evolutionary basal among the *Apis* genus.^14^^,^^17^ *Aflor*OR11 gene and protein sequences are the most different from the three others, with a percentage of identity that does not exceed 96% and 98%, respectively. This relatedness is also observed by comparing these receptors’ patterns of responses (see Figure 3D). Nevertheless, the structural similarities between these four OR11s are striking, and thus, it was not surprising to observe that OR11 in *A. cerana*, *A. dorsata*, and *A. florea* are all strongly activated by 9-ODA, with similar sensitivities to this ligand.

In *A. mellifera*, a macroglomerulus of the drone antennal lobe is selectively activated by 9-ODA (Sandoz 2006), suggesting that together with the OSNs carrying *Amel*OR11,^54^ it represents a dedicated neural channel for the detection and processing of the queen sex pheromone in this species.^31^ Recent work showed that homologs of this *A*. *mellifera* macroglomerulus (MGb) are found within the ALs of the males of the three other species.^60^ Our observation that all four OR11s respond most strongly to 9-ODA reinforce the idea that the 9-ODA-OR11-MGb communication channel is conserved in the three honeybee lineages (*Micrapis*, *Megapis*, and cavity-nesting) and at least among the four studied species. Its putative function is to allow drones to find and follow the queen within congregation areas.^31^^,^^50^ Its conservation within the *Apis* genus can be explained by the crucial importance for the virgin queen, the most valuable individual of the colony, to be quickly and reliably detected by drones, allowing her to gather enough sperm for the rest of her life in only one or two mating flights.^71^^,^^72^ Because 9-ODA is also part of the queen retinue pheromone,^39^^,^^73^ the evolution of OR11 in the *Apis* genus may also be subject to evolutionary pressures related to queen-worker communication of the queens’ fertility.^74^^,^^75^^,^^76^ In any case, our study supports the idea that the last common ancestor of extant honeybees may have possessed a receptor tuned to 9-ODA, a pheromone considered to be an invention of the Apini (honeybees) among other hymenopteran’s fertility signals.^76^ An interesting further research avenue would be to study the co-evolution of the queen fertility signal/sex pheromone and of receptors related to *Apis* OR11 in other tribes of the Apinae.

It is worth noting that the four species studied here are sympatric in many parts of Southeast Asia, as for instance carefully documented in Borneo.^25^^,^^26^^,^^77^ Sexual selection plays a crucial role in evolution, as slight qualitative and quantitative changes in pheromone blends can lead to reproductive isolation and speciation.^3^^,^^11^^,^^78^ However, all *Apis* species produce 9-ODA within their queen mandibular pheromone blend,^40^^,^^41^^,^^44^ and, as discussed earlier, our results suggest that they may exhibit the same neural pathway for 9-ODA detection and processing. This means that this sex pheromone communication channel alone probably does not contribute to pre-mating isolation between these species. Concerning olfactory communication, a number of clues suggest that 9-ODA may not be the only pheromone involved in honeybee mating and that different compounds may be involved in the different species. First, the composition of the mandibular glands differs in the different *Apis* species.^40^^,^^41^^,^^42^^,^^43^^,^^44^ Then, overlapping but different sets of ORs are overexpressed in the antennae of drones compared to the workers of each species.^54^^,^^55^^,^^56^^,^^58^^,^^79^ Lastly, the composition of the drone antennal lobe in macroglomeruli is also different in the four species, from two in open-nesting species to 3–4 in cavity nesting species.^60^ A number of non-olfactory mechanisms are also thought to maintain reproductive isolation among honeybee species such as different genitalia shapes, incompatibilities for sperm storage, as well as different daily periods of sexual activity and different locations of drone congregation areas.^25^^,^^77^^,^^80^

Among the four OR11s, that of *Apis florea* generally displayed lower responses than the other receptors at high doses of 9-ODA (see results for details). Although this effect could theoretically be related to differences in OR11 expression within the *Drosophila* OSNs, we believe it unlikely given the close similarity in the different OR11 sequences and rather lean toward a specificity of the receptor itself. A possible reason for the lower response of *Aflor*OR11 could relate to the putative location of the binding pocket, which appears to be deeper in the receptor than for the three other OR11s, and to involve different residues and in smaller number. This deeper binding pocket may alter the interaction between the residues and the ligand, especially in the second extracellular loop that has been suggested to form a lid over the binding pocket and thus to be involved in the binding between the ligand and the receptor.^81^ The lower response of *Aflor*OR11 could also be relevant from an ecological perspective. Although 9-ODA is known to attract *A. florea* drones, it may play a less important role than in the other species. First, the proportion of 9-ODA found in the mandibular glands of *A. florea* virgin queens is lower, about 15% of the pheromone blend compared to a proportion between 73% (*A. mellifera)* and 91% (*A. cerana*) in the other species*.* Thus, 9-ODA might not be the main pheromone involved in *A. florea* mating.^40^ Interestingly, another compound, 10-HDA, is present in high amounts in the queen mandibular glands of *A. florea* (about 77%) and was shown to attract more dwarf honeybee drones than 9-ODA.^46^ It is thus possible that one of the four other male-biased receptors of *A. florea* (*Aflor*OR18, *Aflor*OR170P, *Aflor*OR155, *Aflor*OR162) detects this compound and plays a central role in this species’ mating behavior.

The four queen pheromone receptor orthologs exhibited responses to secondary ligands. It is generally accepted that sex pheromone receptors are extremely narrowly tuned and should not be triggered by general compounds present in the environment.^54^^,^^82^^,^^83^^,^^84^^,^^85^^,^^86^ To test the specificity of the different OR11s, we performed here a broad screening with floral compounds. As expected, no responses were observed from the four orthologs to the 15 essential oils, which are composed of 21–49 different compounds from different families (ester, terpenes, aldehydes, oxides, ketones, etc.). Nevertheless, a second broad panel of odorants was also tested, including the four components of the *A. mellifera* QMP as well as compounds that shared a structural similarity with 9-ODA and were suggested to induce activity from male *Apis mellifera* placodes responding to 9-ODA.^87^ Again, very few odorants elicited activation of the ORs: *Acer*OR11 displayed significant responses to three new compounds, *Ador*OR11 to eight compounds, and *Amel*OR11 to two compounds. *Aflor*OR11 responded to no other compound than 9-ODA. An intriguing variation in response spectra is thus observed among the different orthologs, with differences in tuning and sensitivity. Although mutations between the four OR11s are not located within residues of the binding pocket, their effect on receptor conformation might impede pore opening and/or ion channel activity and may thus be involved in the differences in affinity and sensitivity observed with some of the secondary ligands. Two ligands, *trans*-2-hexenoic acid and 2-oxovaleric acid, were shared among three orthologs (*Acer*OR11, *Ador*OR11, and *Amel*OR11). *Amel*OR11 was less sensitive to these ligands than *Acer*OR11 and *Ador*OR11 and exhibited a narrower tuning breadth than these receptors. We did not find any obvious ecological relevance for the secondary ligands at this stage, but the possibility exists that they are involved in the reproductive isolation of the four honeybee species since many compounds involved in honeybee mating still remain unknown.^31^ A puzzling case is heptanoic acid, which only activated OR11 in *Apis dorsata*, the giant honeybee (*Megapis* lineage). Conversely, it is possible that the secondary ligands only triggered responses of OR11 by sharing similar molecular features with 9-ODA, especially *trans*-2-hexenoic acid.

One could reasonably question whether odor-evoked responses observed for these OR11 in the *Drosophila* trichoid system reflect this receptors’ function in the honeybee olfactory system, which includes the natural set of odorant binding proteins and degradation enzymes.^88^^,^^89^^,^^90^^,^^91^^,^^92^ We tested whether responses to two of the secondary ligands can be observed in the antennal lobe of *Apis mellifera* males. Using *in vivo* calcium imaging, we observed clear responses to *trans*-2-hexenoic acid within MGb (called MG2 in *A. mellifera*), the macroglomerulus that responds to 9-ODA (Sandoz 2006) and is thought to receive axonal processes from OSNs expressing OR11. We observed some activity in response to 2-oxo-valeric, which was not significant but appeared proportional to the low responses observed in transgenic flies carrying *Amel*OR11. This result confirms the evidence of responses to secondary ligands in the queen-pheromone communication channel and validates the strategy used to study honeybee olfactory receptors in a heterologous system such as the fruit fly.

### Conclusion

As in most species, mating is a very critical moment of honeybees’ life cycle. This complex behavior is highly conserved within the genus *Apis*, in which precious virgin queens fly out of the hive, in open-airspace accessible to predators, and robustly attract males.^71^ Together with the observation of a conserved macroglomerulus in *Apis*,^60^ our study supports the conservation of the main queen sex pheromone channel in the genus, dedicated to the detection and processing of 9-ODA. However, this work also demonstrated the existence of other compounds that can activate the neuronal pathway corresponding to olfactory receptor OR11 in three species of the genus *Apis*, and although close, they do vary across species. The next step would be to investigate whether these compounds are as attractive to drones as 9-ODA, using behavioral experiments in the lab^93^^,^^94^ as well as attraction assays on drone flight corridors^95^ or at congregation areas.^45^^,^^50^^,^^96^^,^^97^ In addition, as a similar mating behavior and attraction of males toward 9-ODA is reported for all the nine accepted species within the genus *Apis*, it would be interesting to search for orthologs of OR11 within their genomes, determine their odorant response spectra, and search for calcium activation within the macroglomeruli of their males’ antennal lobe. Our prediction is that these species probably all share a conserved neuronal pathway dedicated to the processing of 9-ODA, as we observed for *A. cerana*, *A. dorsata*, *A. florea*, and *A. mellifera* in this work. Future studies will also indicate if these sex communication channels also display responses to secondary ligands, more or less specific according to their evolutionary history.

### Limitations of the study

We successfully expressed honeybee OR11 olfactory receptors in olfactory sensory neurons from *Drosophila melanogaster* trichoid sensilla and recorded clear and robust responses from all of them. However, we could not ascertain that expression levels were identical for all OR11 orthologs, nor could we determine how they assembled with *Dmel*ORCO and localized within the neurons. Therefore, the observed differences in the intensity of odorant responses may be due to variations in expression levels and/or membrane targeting of the different OR11 orthologs in the olfactory sensory neurons.

We described the responses of the different OR11 orthologs to 9-ODA as well as to a range of secondary ligands but could not confirm these responses *in vivo* in species other than *A. mellifera*, by optical imaging. Apis species exhibit distinct nesting behaviors that influence their habitat preferences and consequently the ease to study them. The western honeybee (*Apis mellifera*) and the Asian honeybee (*Apis cerana*) are cavity-nesting species, making them amenable to domestication and maintenance in managed hives. This trait has facilitated extensive neuroethological research in these species, including *in vivo* calcium imaging of the male antennal lobe in *A. mellifera* (Sandoz 2006, this study). Conversely, *Apis dorsata* and *Apis florea* are open-nesting bees that present significant challenges for researchers. *A. dorsata*, in particular, build their nests on elevated cliffs, trees, or buildings, complicating efforts to locate and conduct behavioral and neurophysiological experiments on these bees. Developing neurophysiological recordings, such as *in vivo* calcium imaging, in *A. dorsata*, *A. florea*, and *A. cerana* would provide valuable insights into their neurobiology and behavior, advancing our understanding of these ecologically important insects.

## Resources availability

### Lead contact

Further information and requests for resources and reagents should be directed to and will be fulfilled by the lead contact, Jean-Christophe SANDOZ (jean-christophe.sandoz@universite-paris-saclay.fr).

### Materials availability

Further information and requests for resources and reagents should be directed to and will be fulfilled by the lead contact.

### Data and code availability

• The published article and supplemental information include all data generated and analyzed during this study.
• The original code for *in vivo* calcium imaging recordings can be obtained by contacting the lead contact.
• Any additional information required to reanalyze the data reported in this paper is available from the lead contact upon request.

## Acknowledgments

We thank Virginie Larcher for her assistance with the genotyping of transgenic fruit flies and Benjamin Andreu for providing essential oil compound lists shown in Table S1. The study was supported by the 10.13039/501100001665ANR (project ANR-17-CE20-003 to J.C.S.). J.M. received a PhD grant from the French Research Ministry and additional funding from the 10.13039/501100002915Fondation pour la Recherche Médicale (FDT202012020727).

## Author contributions

J.M., T.L., N.M., T.C., and J.C.S. conceived the experiments. J.M., E.L., I.S., and J.C. collected the data. C.M. and J.M. performed protein modeling. J.M., J.C., and J.C.S. analyzed the data and interpreted the results. J.M. and J.C.S. wrote the manuscript. All authors edited the manuscript and approved its final version.

## Declaration of interests

The authors declare no competing interests.

## STAR★Methods

### Key resources table

**Table:** 

REAGENT or RESOURCE	SOURCE	IDENTIFIER
**Bacterial and virus strains**		

pUAST.attB	Synbio Technologies, Monmouth Junction, NJ, USA.	RRID:DGRC_1000

**Biological samples**		

Fura-2 dextran	Life technologies, Saint-Aubin, France	F3029
Tetramethylrhodamine dextran	Life technologies, Saint-Aubin, France	D3307

**Chemicals, peptides, and recombinant proteins**		

9-oxo-decenoic acid (9-ODA)	Apollo Scientific ltd., Bredbury, United Kingdom	CAS 334-20-3
9-hydroxydecenoic acid (9-HDA)	Sigma Aldrich, Saint-Louis, MO, USA	CAS 1422-27-1
methyl 4-hydroxybenzoate (HOB)	Sigma Aldrich, Saint-Louis, MO, USA	CAS 99-76-3
homovanillyl alcohol (HVA)	Sigma Aldrich, Saint-Louis, MO, USA	CAS 2380-78-1
cis-2-hexen-1-ol	Sigma Aldrich, Saint-Louis, MO, USA	CAS 928-94-9
trans-2-hexen-1-ol	Sigma Aldrich, Saint-Louis, MO, USA	CAS 928-95-0
6-methyl-5-hepten-2-one	Sigma Aldrich, Saint-Louis, MO, USA	CAS 110-93-0
trans-2-hexenal	Sigma Aldrich, Saint-Louis, MO, USA	CAS 6728-26-3
heptanoic acid	Sigma Aldrich, Saint-Louis, MO, USA	CAS 111-14-8
hexanoic acid	Sigma Aldrich, Saint-Louis, MO, USA	CAS 142-62-1
isovaleric acid	Sigma Aldrich, Saint-Louis, MO, USA	CAS 503-74-2
lauric acid	Sigma Aldrich, Saint-Louis, MO, USA	CAS 143-07-7
4-methyl-pentanoic acid	Sigma Aldrich, Saint-Louis, MO, USA	CAS 646-07-1
myristic acid	Sigma Aldrich, Saint-Louis, MO, USA	CAS 544-63-8
2-oxooctanoic acid	Sigma Aldrich, Saint-Louis, MO, USA	CAS 328-51-8
2-oxovaleric acid	Sigma Aldrich, Saint-Louis, MO, USA	CAS 1821-02-9
propionic acid	Sigma Aldrich, Saint-Louis, MO, USA	CAS 79-09-4
trans-2-hexenoic acid	Sigma Aldrich, Saint-Louis, MO, USA	CAS 13419-69-7
trans-3-hexenoic acid	Sigma Aldrich, Saint-Louis, MO, USA	CAS 1577-18-0
tridecanoic acid	Sigma Aldrich, Saint-Louis, MO, USA	CAS 638-53-9
undecanoic acid	Sigma Aldrich, Saint-Louis, MO, USA	CAS 112-37-8
*Eucalyptus globulus* (essential oil)	Aromatics International, Paris, France	EUP-107
*Eugenia caryophyllata* (essential oil)	Aromatics International, Paris, France	CLB-113
*Cymbopogon martinii var. motia* (essential oil)	Aromatics International, Paris, France	PRS-115
*Mentha citrata* (essential oil)	Aromatics International, Paris, France	BMM-110
*Zingiber cassumunar* (essential oil)	Aromatics International, Paris, France	PLN-108
*Citrus aurantium var. Amara or Bigaradia* (essential oil)	Aromatics International, Paris, France	PGA-108
*Myrtus communis* (essential oil)	Aromatics International, Paris, France	MRG-103
*Foeniculum vulgate* (essential oil)	Aromatics International, Paris, France	FEN-106
*Daucus carota ssp. sativus* (essential oil)	Aromatics International, Paris, France	CRS-111
*Matricaria recutita* (essential oil)	Aromatics International, Paris, France	GCE-102
*Chamaemelum nobile* (essential oil)	Aromatics International, Paris, France	RCM-117
*Curcuma longa* (essential oil)	Aromatics International, Paris, France	TUM-108
*Abies sibirica* (essential oil)	Aromatics International, Paris, France	PNE-117
*Hypericum perforatum* (essential oil)	Aromatics International, Paris, France	SJW-103
*Jasminum grandiflorum* (essential oil)	Aromatics International, Paris, France	JSA-114

**Experimental models: Organisms/strains**		

*Drosophila melanogaster* y1 M[vas-int.Dm]ZH-2A w∗; M[3xP3-RFP.attP]ZH-51C	BestGene Inc. (Chino Hills, CA, USA)	www.thebestgene.com/
*Drosophila melanogaster* X-OR11	This paper	N/A
*Drosophila melanogaster* DmelOR67d^GAL4^	Kurtovic et al.^66^	N/A
*Apis mellifera ligustica*	This paper	RRID:NCBITaxon_7469

**Software and algorithms**		

dbWave (2020)	Marion Poll and Tobin^98^	N/A
GraphPad Prism (9)	This paper GraphPad Software, San Diego, CA, USA	www.graphpad.com
R Software (4.3.2)	RCoreTeam^99^	www.r-project.org
T.I.L.L. VisION (4.0)	Martinsried, Germany	www.support.moleculardevices.com
Visiview (3.3.0.0)	Visitron Systems, Puchheim, Germany	www.visitron.de
IDL (6.0)	Research Systems Inc., Colorado, USA	www.vision-systems.com
Excel (2019)	Microsoft, Redmond, USA	www.microsoft.com

### Experimental model and study participant details

#### Fruit fly experiments

In all experiments, the flies (genotypes: *w; UAS-X-OR11; Or67d*^*GAL4*^*x w; UAS-GCaMP6m; Or67d*^*GAL4*^*)* were tested between 3 and 7 days old, and in each experiment, males and females were used in a 50-50 ratio. Flies were kept in a controlled environment (temperature: 25°C, 12h light: 12h dark daily cycle) and fed on axenic yeast food. Fruit flies of the same sex from the same breeding environment were randomly assigned to experimental groups.

#### Honey bee experiments

*In vivo* calcium imaging experiments were performed only on male honey bees (Western honey bee, *Apis mellifera ligustica*) as the study addresses the detection of sex pheromones by the drones. They were collected at a hive entrance, on sunny summer days, between 3.00 pm and 5.00 pm. The males flying out of the hives at these times (between 3.00 and 5.00 pm) are older than 12 days.^26^ The hives are kept in an open environment on the Saclay plateau in Gif-sur-Yvette, at the IDEEV institute.

### Method details

#### Study design

This study employs a range of experimental approaches. Transgenic flies expressing honey bee OR11 genes within ab3 basiconic sensilla OSNs were prepared, and were validated in a first experiment employing single-sensillum recordings of ab3 sensilla. Then, we used transcuticular calcium imaging of the drosophila antenna^67^ to apply a screening approach and search for OR11 ligands. First, we conducted tests with essential oils, thereby offering a broad spectrum of potential floral ligands. Second, we examined a series of odorants produced by the queen mandibular glands as well as compounds exhibiting chemical similarity to the sex pheromone 9-ODA. Thanks to these screening experiments, we identified secondary ligands and assessed the sensitivity of OR11 receptors to 9-ODA and these secondary ligands by establishing dose-response curves. Finally, we directly tested these odorants on the male *A. mellifera* antennal lobe using *in vivo* calcium imaging.

#### 3D molecular prediction of OR11 proteins structure

Alignment between the protein sequences of the four OR11s was made with MAFFT as implemented in JalView.^100^ The 3D molecular structure prediction was done using AlphaFold2^101^ with AMBER relaxation on the Institut Français de Bioinformatique (IFB) Core Cluster (ANR-11-INBS-0013) using the protein sequences of the different OR11 orthologs deduced from previously identified coding sequences.^54^^,^^58^^,^^102^^,^^103^ Coordinates of the binding pocket were computed using DeepSite (https://playmolecule.com/deepsite/)^104^ and docking prediction was performed using Webina (https://durrantlab.pitt.edu/webina/).^105^ Residues located near the ligand and that can form hydrogen bonds were identified using ChimeraX. Mapping of the protein and ligand models were performed using PyMOL.^106^

#### Fly genetics

The full-length open reading frames encoding OR11 in four honey bee species (*Apis cerana, A. dorsata, A. florea, A. mellifera;* Wanner et al.,^54^ Jain and Brockmann,^55^ Karpe et al;^58^ Oppenheim et al;^102^ Park et al;^103^), respectively called *Acer*OR11, *Ador*OR11, *Aflor*OR11 and *Amel*OR11 were synthesized *in vitro* with codon optimization for expression in *Drosophila*, and then sub-cloned into the pUAST.attB vector (Synbio Technologies, Monmouth Junction, NJ, USA). Balanced transformant *UAS-X-OR11* fly lines were generated by BestGene Inc. (Chino Hills, CA, USA) by injecting the pUAST.attB-OR11 plasmids into fly embryos of genotype *y1 M[vas-int.Dm]ZH-2A w∗; M[3xP3-RFP.attP]ZH-51C*, leading to a non-random insertion of the *UAS-X-OR11* construct into locus 51C of the second chromosome.^107^ Subsequently, we crossed the *UAS-OR11* lines with a *DmelOr67d*^*GAL4*^ mutant line to induce OR11 expression within at1 OSNs as replacement for the endogenous odorant receptor *Dmel*OR67d.^66^ By doing so, we created four homozygous *w; UAS-X-OR11; Or67d*^*GAL4*^ lines. Finally, for transcuticular calcium imaging experiments, homozygous *w; UAS-X-OR11; Or67d*^*GAL4*^ lines were crossed with homozygous *w; UAS-GCaMP6m; Or67d*^*GAL4*^ flies.^67^ The presence of the *UAS-X-OR11* transgenes was successfully verified using PCR on genomic DNA extracted from 5 flies (Primers: Forward: CCAGCTGGATCAGTTCGTGC; Reverse: GCAAGTCACTTGGAACACC).

Flies were kept in a controlled environment (temperature: 25°C, 12h light: 12h dark daily cycle) and fed on axenic yeast food.

#### Olfactory stimulations

The odorant cartridges were prepared with glass Pasteur pipettes containing each a 1 cm^2^ piece of filter paper to which 5 μL of odorant solution were applied, and 1 mL plastic pipette tips, which were placed on the open end of each pipette. New stimulation cartridges were prepared each day.

For Single Sensillum Recording (SSR) and the first transcuticular calcium imaging experiment on *Amel*OR11, *Acer*OR11, *Ador*OR11 and *Aflor*OR11, 3 different odorants were used: the honey bee queen pheromone, 9-oxo-(E)-decenoic acid (9-ODA), and two controls (the solvent, 2-propanol, and cVA). cVA is the ligand of the endogenous *Drosophila Dmel*OR67d receptor, and was used to check that flies no longer express this receptor.

Olfactory stimulations used in the first broad screening experiment were essential oils diluted to 10^-2^ in mineral oil (Aromatics International, Florence, United States). We tested 15 essential oils, chosen for the diversity of chemical compounds they contain (Table S1). Mineral oil was presented as control.

For the second screening experiment, we selected compounds present in the Queen Mandibular Pheromone of the honey bee *Apis mellifera*: 9-ODA (Apollo Scientific ltd., Bredbury, United Kingdom), 9-HDA, HOB and HVA (Sigma-Aldrich, St. Louis, Missouri, United States). Some of these compounds are also present in the QMPs of the other Apis species^40^^; see introduction^. These pheromones were tested at 250 μg (5 μL of a 50 μg.μL^-1^ dilution on filter paper). We also tested 18 odorants (Sigma-Aldrich, St. Louis, Missouri, United States) selected from the study by Vareschi^87^ in which these odorants appear to potentially activate the same neural units as 9-ODA in electrophysiological recording of placoid sensilla. We tested these odorants at 100 μg (10 μL on filter paper from 10 μg.μL^-1^ dilution) (Table 1). All these compounds were diluted in 2-propanol, which was also presented as a control.

Lastly, for *in vivo* calcium imaging recordings on honeybee drones, 7 odorants were used: QMP compounds at 250 μg (9-ODA, 9-HDA, HOB, HVA), two secondary ligands of OR11 (see results), trans-2-hexenoic acid and 2-oxo valeric acid at 100 μg on filter paper as in the fly experiments. The solvent, 2-propanol, was also tested.

In each experiment, odorants were presented in random order, except for the dose-response experiments in which the odorants were tested at increasing doses. In these experiments, we tested three odorants, all diluted in 2-propanol: 9-ODA, trans-2-hexenoic acid and 2-oxo-valeric acid. They were all tested at doses from 0.1 μg to 1000 μg, increasing logarithmically (doses 0.1, 0.3, 1, 3, 10, 30, 100, 300 and 1000 μg were tested). In all experiments, the interval between stimulations was 2 minutes.

#### Transcuticular calcium imaging recordings of the drosophila antenna

Three to 7-day-old flies were immobilized in an ABS (Acrylonitrile-butadiene-styrene) plastic chamber with only the head protruding. Their wings were fixed using myristic acid to avoid fly body movement. The fly’s antennae were constrained with a thin piece of Parafilm® (Bemis Company, Inc., Neenah, Wisconsin, USA) in order to access the region of the antenna where the at1 sensilla are located.^108^ Recordings were performed using dedicated routines under Visiview 3.3.0.0 software and were performed with a 10x water-immersion objective (Olympus UMPlanFI 10x/0.30 W) on an epifluorescence microscope (Olympus BX-51WI) coupled with an EMCCD-camera (Evolve™ 512, Photometrics). A monochromator produced an excitation light at 488 nm (Polychrome 5000). Each recording consisted in 100 frames at a frequency of 5 Hz (20 s recording), with an exposure time of 80 ms. A constant airstream (3 L/min) was directed from a distance of 1 cm towards the antennae of the fly. Olfactory stimulation lasted between the 15^th^ frame (i.e. after 3 s) and the 20^th^ frame (1 s stimulation). For the duration of the stimulus, part of the main air flow (500 mL/min) was redirected from an empty pipette to the stimulation pipette. Thus, the airflow reaching the fly was constant. In order to correct for photobleaching of the calcium reporter (see below), a few recordings without any stimulus were performed, allowing to measure GCaMP6m fluorescence decay during the 100 frames of a recording. One of these recordings was used for photobleaching correction.

We used VisiView 3.3.0.0 software to extract the data. Regions of interest (ROI) were manually drawn around each antenna, and the average fluorescence level within each ROI at each frame was exported. Fluorescence changes over time were calculated using the equation ΔF/F_0_ = (F-F_0_) / F_0,_ where F_0_ is the mean fluorescence value over 5 frames before the stimulation (frames 10 to 14) and F the fluorescent at frame n. For each fly, the curve measuring GCaMP6m fluorescence decay over time was subtracted from all other curves to correct for photobleaching. Response amplitudes were calculated by the difference between F_0_ and the average of 15 frames after the stimulation (frame 17-31) and were achieved on R software (v4.3.2 RCoreTeam^99^) and Microsoft Excel 2013.

#### *In vivo* calcium imaging recordings of the honey bee antennal lobe

For *in vivo* calcium imaging experiments, *Apis mellifera* males (drones) were collected at a hive entrance, on sunny summer days, between 3.00 pm and 5.00 pm, when mature males (usually older than 12 days) leave the hive to join mating congregations. Individual drones were placed on ice until they stopped moving and were then fixed in a recording chamber using low-temperature melting wax. A window was then cut in the cuticle of the head, between the compound eyes, ocelli and base of the antennae. Trachea and membranes were removed to expose the brain, which was immersed in saline solution (in mM: NaCl, 130; KCl, 6; MgCl_2_, 4; CaCl_2_, 5; sucrose, 160; glucose, 25; HEPES, 10; Ph 6.7, 500 mOsmol; all chemicals from Sigma-Aldrich, St-Louis, MO, USA). A pulled glass electrode coated with the calcium indicator Fura-2 dextran (potassium salt, 10.000 kDa, in 2% BSA; Life technologies, California, USA) mixed with tetramethylrhodamine dextran (10.000 kDa; Life technologies, Saint-Aubin, France) was inserted on the axonal path of l-ALT projection neurons, between the α lobe and the optic lobe border, rostrally from the lateral horn.^109^^,^^110^^,^^111^^,^^112^ Drones were then placed into a dark humid box for at least 3 hours, during which the dyes were allowed to migrate retrogradely towards the AL, thereby staining the glomeruli innervated by the l-ALT. Calcium imaging recordings were performed using a T.I.L.L. Photonics imaging setup (Martinsried, Germany) as in previous studies.^112^^,^^113^ Stained drones were placed under the 10x water-immersion objective (Olympus, UMPlanFL; NA 0.3) of an epifluorescent microscope (Olympus BX-51WI). Recordings were done using a 640 x 480 pixel 12-bit monochrome CCD-camera (T.I.L.L) with 4 x 4 binning on the chip (pixel image size: 4.8 x 4.8 μm). Alternated excitation at 340 nm and 380 nm monochromatic light (T.I.L.L Polychrom IV) was applied as 100 double frames at a frequency of 5 Hz. A 490 nm dichroic filter and a bandpass 525/50 nm emission filter were used to detect fluorescence. Integration time was 4-20 ms at 380 nm and 16-80 ms at 340 nm excitation. The odorant stimulation was applied between the 15^th^ and the 20^th^ frames, and thus lasted 1 s. The olfactory stimulation system was similar to that used in the transcuticular calcium imaging experiments.

Honey bee *in vivo* imaging data were analyzed using IDL 6.0 (Research Systems Inc., Colorado, USA) as in previous studies.^112^^,^^113^ Each recording corresponded to a 4-dimensional array with the excitation wavelength (340 nm or 380 nm), two spatial dimensions (x, y pixels of the area of interest) and the temporal dimension (100 frames). For data analysis we applied three steps following Galizia and Vetter.^114^ First, the ratio *R = F340 nm/F380 nm* was calculated at each pixel and time point. Then, we computed relative ratio changes defined by *ΔR/R = (R-R0)/R0*, with the average of five frames before the start of any olfactory stimulation (frames 10–14) as reference R0. Data were then filtered in both spatial dimensions and in the temporal dimension using a three-pixel median filter in order to reduce the effects of photon and electronic noise. At last, a bleach correction was applied. For each recording, a logarithmic curve fitted to the median brightness decay of the entire image frames, excluding the frames during the stimulus until 5 s after stimulus onset, was subtracted from the data (Galizia and Vetter, 2004). Response amplitude was measured as the mean of three frames during the stimulus (frames 17–19) minus the mean of three frames before the stimulus (frames 9–11).

### Quantification and statistical analysis

Statistical analysis for all experiments was performed using GraphPad Prism 9. For each dataset, normality was verified using the Shapiro-Wilk test. Depending on normality, parametric or non-parametric statistical tests were used. SSR data followed a normal distribution and were thus analyzed using ANOVA for repeated measurements (RM-ANOVA), with Greenhouse-Geisser correction. Pairwise comparisons were done using Dunnett’s multiple comparison test, comparing the response to each odorant to its respective solvent control. As transcuticular calcium imaging data did not follow a normal distribution, the Friedman test was used to compare response amplitudes between stimuli, and post-hoc comparisons were performed using Dunn’s corrected multiple comparison post hoc tests. Most of the data of dose-responses analyses followed normal distributions and were thus analyzed using RM-ANOVA with Geisser-Greenhouse correction, and pairwise comparisons using a corrected Dunnett’s multiple comparison test. Dose-response analyses that did not follow a normal distribution were compared using a Friedman Test and Dunn’s corrected multiple comparison test. All comparisons between different OR11s were performed using a Kruskal-Wallis test followed by Dunn’s corrected multiple comparison tests. For dose-response curves, EC_50_ and Hill coefficient were calculated using GraphPad Prism 9. They were compared among OR11 using the Kruskal-Wallis test.

Data normalization and heatmaps were made using GraphPad Prism 9 (GraphPad Software, San Diego, CA, USA), by considering the 9-ODA response as the maximum and solvent response as the minimum. Normalization of dose-response curves for the two secondary ligands was performed by taking the 9-ODA maximum responses at the maximum dose as a 100% response.

Using the data obtained with the second panel of odorants (Table 1), we computed a measure of the specificity of each receptor, from the distribution of its response amplitudes. It is referred to as *sparseness*, using the formula from Rolls and Tovee^68^:

$$S=\left(\frac{1}{1-\frac{1}{n}}\right)\times \left(1-\frac{{\left(\sum_{i=1,n}r_{i}/n\right)}^{2}}{\sum_{i=1,n}\left(r_{i}^{2}/n\right)}\right)$$

With *r*_*i*_ being the amplitude of response to stimulus *i* in the set of *n* stimuli. Because this formula cannot compute negative responses, they were set to 0.

Dendrograms were performed on R software (v4.3.2 RCoreTeam^99^).

All statistical analyses are presented in the supplemental information (Table S3).

## Supplemental information

Table S1. Description of the panel of essential oils shown in Figure 3A

Table S2. Binding pocket coordinates for each OR11 protein model

Table S3. Raw data and statistical analysis for all experiments (A) Raw data for the first test using single-sensillum recording of *Amel*OR11 shown in Figures 1A and 1B. (B) Statistical analysis for the first single-sensillum recording of *Amel*OR11 (Figure 1C). (C) Raw data and statistical analysis for the first transcuticular calcium imaging test with *Amel*OR11 (Figures 1D–1F). (D) Statistical analysis of calcium imaging responses to the essential oil panel for the four OR11 receptors (Figure 3A). (E) Raw data of transcuticular calcium imaging responses to the essential oil panel for the four OR11 receptors (Figure 3A). (F) Statistical analysis of transcuticular calcium imaging responses to the broad Vareschi (1971) panel for the four OR11 receptors (Figure 3B). (G) Raw data of transcuticular calcium imaging responses to the broad Vareschi (1971) panel for the four OR11s (Figure 3B). (H) Statistical analysis of transcuticular calcium imaging responses, comparison between the four OR11 receptors responses to the broad Vareschi (1971) panel (Figure 3B). (I) Raw data of responses to the broad Vareschi (1971) panel for the control fruit fly line (without OR expression in trichoid sensilla). (J) Raw data of dose-response curves for the four OR11 receptors (Figure 4C). (K) Comparative statistical analysis of dose-response curves for the four OR11s receptors (Figure 4C). (L) *In vivo* calcium imaging raw data (Male *Apis mellifera*) and statistical analysis (Figure 5).
